# Parvovirus B19 Infection in Adults: A Case Series

**DOI:** 10.7759/cureus.63169

**Published:** 2024-06-25

**Authors:** Cundullah Torun

**Affiliations:** 1 Internal Medicine, Istanbul Medeniyet University Göztepe Training and Research Hospital, İstanbul, TUR

**Keywords:** parvovirus b19, polyarthralgia, infection outbreak, skin rash, proximal interphalangeal joint

## Abstract

Parvovirus B19 infection, typically associated with erythema infectiosum in children, presents variably in adults, often leading to misdiagnosis. This case series describes three adult patients diagnosed with parvovirus B19 infection in an internal medicine outpatient clinic in March 2024. Symptoms included fatigue, joint pain, swelling, and skin rash, with misdiagnoses including early rheumatoid arthritis. The diagnosis was confirmed via positive parvovirus antibodies and polymerase chain reaction (PCR). All patients received supportive care, and symptoms resolved within an average of 18 days. This series underscores the need for heightened clinical suspicion and timely serological testing for parvovirus B19 in adults presenting with flu-like symptoms, joint pain, and rash, especially during mini-outbreaks and following contact with infected children.

## Introduction

Parvovirus B19 infection is commonly recognized as erythema infectiosum in children, characterized by a maculopapular rash on the cheeks. In adults, it typically begins with flu-like symptoms and progresses to arthralgia, joint swelling, and skin rash [1]. In addition, adults may experience more severe complications, such as transient aplastic crisis, especially in individuals with underlying hemolytic disorders, and chronic anemia in immunocompromised patients, making early detection crucial to prevent these severe outcomes. Infections with parvovirus B19 are more frequent in late winter, spring, and early summer, with mini outbreaks occurring approximately every three to four years [2]. The virus is transmitted primarily via respiratory droplets, but it can also be spread through blood transfusions and from mother to fetus, potentially leading to hydrops fetalis and fetal death in pregnant women [3]. Secondary transmission to seronegative contacts is common, with a secondary attack rate of 20-30% among susceptible adults in school or household settings during erythema infectiosum epidemics [4]. Despite this, the literature on parvovirus infection in adults is limited to case reports and small studies, and internists treating adult patients may not be fully aware of the clinical features of parvovirus B19 infection [5]. Given the varied clinical presentations and potential for severe outcomes, heightened awareness and consideration of parvovirus B19 in the differential diagnosis of adults presenting with compatible symptoms are crucial. This case series describes the diagnostic process for suspected parvovirus B19 infection in adults presenting with rash and joint pain at the internal medicine outpatient clinic of a tertiary care hospital.

## Case presentation

Case 1

A 38-year-old single woman presented to the emergency department with fatigue and generalized itching. She had no history of chronic illness, medication use, recent travel, or animal contact, and there was no history of suspicious sexual encounters. Initial treatment included oral antihistamine (rupatadine 10 mg). Laboratory tests indicated elevated transaminase levels, approximately three times the normal range (0-40 U/L), resulting in a referral to the internal medicine outpatient clinic.

Five days later, she visited the clinic reporting swelling in her fingers, a rash on her upper and lower limbs, and widespread joint pain. Her vital signs were normal: temperature 36.5°C, blood pressure 114/70 mmHg, heart rate 71 bpm, and respiratory rate 15/min. Physical examination revealed tenderness and edema in the proximal interphalangeal (PIP) and metacarpophalangeal joints. A hepatobiliary ultrasound showed no pathology. She had no chronic illnesses and had received all routine childhood vaccines, including the measles-mumps-rubella vaccine. Serologies for hepatitis B and C viruses and HIV were negative.

Given her profession as a kindergarten teacher and a recent fifth disease outbreak among her students, parvovirus B19 antibodies were tested. Both IgM and IgG were positive, with a high PCR result (203600). Apart from elevated transaminase levels and mild anemia, no other significant laboratory findings were noted. Supportive treatment, including rest, increased fluid intake, and nonsteroidal anti-inflammatory drugs (NSAIDs) as needed, was provided. When she returned 20 days after symptom onset, her symptoms had mostly resolved, except for mild pain in the PIP joints.

Case 2

A 25-year-old married woman, who had recently traveled to her hometown two months prior and kept a cat at home, presented with fatigue, generalized muscle pain, and a subfebrile fever (37.8°C). She had no known illnesses or regular medication use. Her initial symptoms began 10 days earlier, and she managed them at home with paracetamol. A week after symptom onset, she developed severe joint pain and swelling in her fingers, wrists, and ankles, leading to a preliminary diagnosis of early rheumatoid arthritis at a rheumatology clinic.

Upon examination at the clinic, she had bilateral pain and edema in the PIP joints. Vital signs were normal: temperature 37.2°C, blood pressure 110/65 mmHg, heart rate 72 bpm, and respiratory rate 14/min. Laboratory tests showed no significant findings except for positive parvovirus antibodies (IgM >200, IgG >50, PCR 121200). Brucella (Wright tube agglutination) and toxoplasma IgM tests were negative. Supportive treatment was provided. Her symptoms completely resolved 17 days after the onset.

Case 3

**Figure 1:**
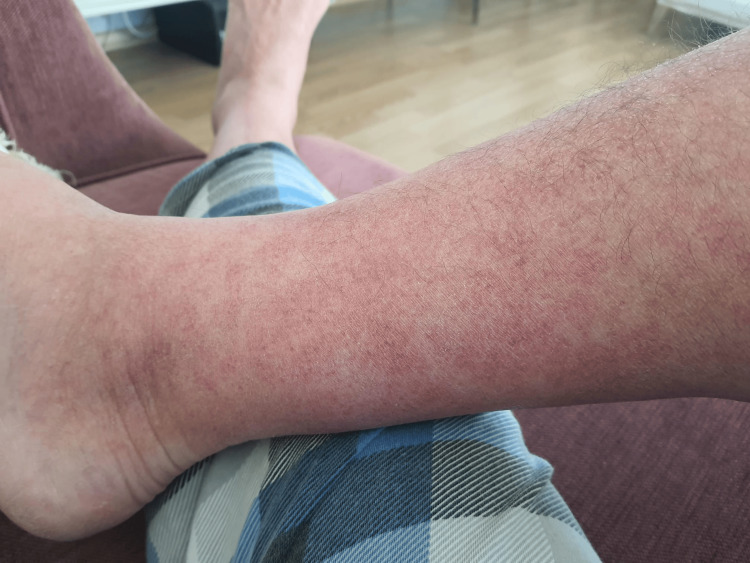
The picture shows a diffuse erythematous rash covering a significant part of the lower leg.

A 61-year-old man with ankylosing spondylitis, treated with colchicine, reported fever (38.4°C), fatigue, and muscle pain starting two weeks before his visit to my clinic, followed by an erythematous rash on the legs (Figure 1), swelling, and pain in the interphalangeal joints. He experienced alternating diarrhea and constipation. By the time he visited my clinic, his prodromal symptoms and rash had subsided. His vital signs were within normal limits: temperature 36.8°C, blood pressure 120/78 mmHg, and heart rate 76 bpm. Physical examination revealed ongoing swelling and pain in the PIP joints. He had no recent travel history or animal contact. He was taking care of her grandson at home and that there was a fifth disease outbreak recently at his grandson's school. Laboratory findings were generally unremarkable except for positive parvovirus antibodies (IgM >200, IgG 42.3, PCR 71510) and mild anemia. Supportive treatment was continued, and his symptoms gradually improved over the following weeks. Seventeen days after the onset of his complaint, his physical examination was completely normalized, but the pain in the bilateral proximal interphalangeal joints was still persistent at the end of one month.

The clinical and laboratory characteristics of the patients are presented in Table 1. All three patients received supportive treatment, with the third patient also receiving nonsteroidal anti-inflammatory drugs (NSAIDs). Symptoms resolved on average 18 days after onset, and liver function tests normalized in the first patient.

**Table 1:** Clinical and laboratory characteristics of the patients Ig: immunglobulin; PCR: polymerase chain reaction; WBC: white blood cell count; HGB: hemoglobin; AST: aspartate transaminase; ALT: alanine transaminase

	Case 1	Case 2	Case 3	Normal Range
Age	38	25	61	
Gender	Female	Female	Male	
Arthralgia	+	+	+	
Joint swelling	+	+	+	
Rash	+	-	+	
IgM	>200	>200	>200	(0-20)
IgG	>50	>50	42.3	(0-2)
PCR	203600	121200	71510	(<1)
WBC (/mL)	5400	4300	7.6	(4000-10000)
HGB (g/dL)	11.4	12.1	12.5	(13-17)
AST (U/L)	65	11	15	(0-40)
ALT (U/L)	191	18	23	(0-41)

## Discussion

In this case series, three adult parvovirus cases diagnosed within a one-month period in an internal medicine outpatient clinic are presented, highlighting their diverse demographic characteristics and clinical courses, which led to repeated hospital visits with different preliminary diagnoses.

Parvovirus infection, typically presenting with a maculopapular rash in children, can manifest with various symptoms in adults, making it less likely to be considered by primary care providers, such as family physicians and internists. Consequently, adult patients with parvovirus B19 infection may not be accurately diagnosed and might be referred to other specialties like rheumatology, hematology, or dermatology. The literature includes many cases of parvovirus infection being initially misdiagnosed as conditions like lupus erythematosus, myeloproliferative disorders, and even acute myocardial infarction [6-8].

Following intranasal exposure to B19, viremia occurs within four to 14 days, presenting with flu-like prodromal symptoms (first phase) [9]. The onset of anti-B19 antibody clearance marks the end of viremia and the beginning of the second phase, characterized by rash, arthralgias, and arthritis [10]. All three cases in this series exhibited the triad of arthralgia, joint swelling, and, in two cases, skin rash, underscoring the importance of considering parvovirus infection in the differential diagnosis when these symptoms are present.

Experimental studies have shown that primary and secondary symptoms appear in a biphasic manner [9], but symptom timing can vary among patients. In a prospective study by Waza et al., patients over 18 years old with major parvovirus symptoms and contact history were assessed, and 14 patients were diagnosed with parvovirus. Some patients experienced a simultaneous onset of first- and second-phase symptoms, while others had a delay of about a week between the phases [5]. In our series, the second patient had a week-long gap between flu-like symptoms and the second phase, the third patient had nearly simultaneous symptom onset, and the first patient had itching as the primary symptom followed by joint pain and swelling four to five days later.

Unlike the typical facial rash seen in children, adults may present with purpuric generalized or localized rashes [11,12]. The rashes in the first and third cases were purpuric with a distal symmetric distribution. Joint symptoms occur in about 60% of infected adults, more commonly in women (59%) than in men (30%), often presenting as acute-onset symmetric polyarticular arthritis primarily affecting small joints, such as the PIP and MCP joints [13]. All three cases had bilateral small joint involvement, consistent with other reports.

Most patients with parvovirus B19 infection do not require laboratory studies due to the mild nature of the illness, which resolves within five to seven days. Acute or chronic infection may be diagnosed using DNA hybridization or quantitative PCR in combination with serologic assays for B19-specific IgG and IgM [14]. IgM antibodies are detected 10-12 days after inoculation, and IgG antibodies are detected at two weeks. In all three cases, both IgM and IgG were positive due to the antibody tests being conducted at least a week after symptom onset.

## Conclusions

This case series underscores the importance of considering parvovirus B19 infection in adults presenting with non-specific flu-like symptoms, joint pain, swelling, and skin rashes. Heightened clinical awareness and timely serological testing for parvovirus B19 are essential to prevent misdiagnosis and unnecessary referrals to other specialties. Supportive care remains the mainstay of treatment for parvovirus B19 infection in adults, as evidenced by the resolution of symptoms in our patients within an average of 18 days.

Primary care providers and internists should consider parvovirus B19 infection in the differential diagnosis for adults presenting with compatible symptoms, particularly during periods of increased incidence or mini-outbreaks. This proactive approach can significantly improve patient outcomes and reduce healthcare costs associated with misdiagnosis and inappropriate treatments.
